# Optimizing the Simplicial-Map Neural Network Architecture

**DOI:** 10.3390/jimaging7090173

**Published:** 2021-09-01

**Authors:** Eduardo Paluzo-Hidalgo, Rocio Gonzalez-Diaz, Miguel A. Gutiérrez-Naranjo, Jónathan Heras

**Affiliations:** 1 Department of Applied Mathematics I, University of Sevilla, 41012 Sevilla, Spain; rogodi@us.es; 2 Department of Computer Sciences and Artificial Intelligence, University of Sevilla, 41012 Sevilla, Spain; magutier@us.es; 3 Department of Mathematics and Computer Science, University of La Rioja, 26004 Logroño, Spain; jonathan.heras@unirioja.es

**Keywords:** simplicial-map neural networks, artificial neural networks, computational topology

## Abstract

Simplicial-map neural networks are a recent neural network architecture induced by simplicial maps defined between simplicial complexes. It has been proved that simplicial-map neural networks are universal approximators and that they can be refined to be robust to adversarial attacks. In this paper, the refinement toward robustness is optimized by reducing the number of simplices (i.e., nodes) needed. We have shown experimentally that such a refined neural network is equivalent to the original network as a classification tool but requires much less storage.

## 1. Introduction

In spite of the undoubted advantages of deep learning techniques for classification tasks [1], many important problems remain still unsolved in this context. In particular, if we focus on the efficiency of such models, one of their main drawbacks is the huge amount of resources needed for training competitive networks (for instance, vision models based on the transformer architecture need billions of images to be trained [2]). In many cases, only big companies can support the expensive cost of training competitive architectures [3,4]. From a practical point of view, one of the open research lines in deep learning is the exploration of ways to reduce training resources without reducing the accuracy of trained models.

One way to reduce time (to train the model) and space (to store the training data set) is to take a small subset of the training data set that summarizes its useful information. Several authors have explored this idea. For example, in [5], a data set representative of the training data set was considered. In [6], techniques of active learning were applied to classify images using convolutional neural networks. In [7], the authors reduced the volume of the training data set using stochastic methods. Other authors, in [8], replaced the training data set with a small number of synthetic samples containing all the original information.

Another approach tries to reduce the number of training parameters by pruning the model. This is a general technique in machine learning and it has a long tradition in neural networks [9]. The importance of pruning neural networks has emerged in recent years due to the big amount of resources required in deep learning [10,11,12]. Since local search techniques based on back propagation play a central role in weight optimization, the different pruning techniques can be classified using such a training process as the main reference. For example, there are studies where pruning occurs at the end of the training process [13], after the training process [14] or in the early stages of the training process [15].

Topological data analysis (TDA) provides a completely different approach to reducing the number of resources in the neural network classification process. In [16], the authors provided a constructive approach to the problem of approximating a continuous function on a compact set in a triangulated space. Once a triangulation of the space is given, a two-hidden-layer feedforward network with a concrete set of weights called a simplicial-map neural network is computed. The construction is based on several strong theorems from algebraic topology and allows one to avoid the heavy process of optimizing the weights of neural networks since they can compute the weights directly from the triangulation of the space. Later, in [17], the authors showed that simplicial-map neural networks can be defined to be robust to adversarial attacks of a given size.

Simplicial-map neural networks are vaguely related to margin-based classifiers such as support vector machines (SVMs) and to nonparametric methods such as *k*-nearest neighbors (*k*-NN). These algorithms are widely used and, in both cases, there exist efforts to study their robustness to adversarial examples such as [18] in the case of *k*-NN or [19] for SVMs. Simplicial-map neural networks are not trained but defined on a triangulation of the data set and the decision boundaries are based on that triangulation. One of the greatest advantages of this approach is the possibility of formal proof of different properties such as universal approximation ability and, as previously mentioned, robustness against adversarial examples. However, both properties are based on barycentric subdivisions of the triangulation with a large increase in required storage as the number of simplices grows, this being a bottleneck for its applicability.

In this paper, we propose an algorithm to reduce the number of parameters of simplicial-map neural networks without reducing their accuracy. The key to the proposed method is that barycentric subdivisions, in particular, and triangulations of training data sets, in general, introduce many simplices that are not needed or redundant. The paper is organized as follows. In Section 2, we recall some basic concepts. In Section 3, we provide the description of our methodology. The description is illustrated with some examples in Section 4. We finish the paper with some conclusions and hints for future work.

## 2. Background

In [16,17], a new approach to construct neural networks based on simplicial maps was introduced. Roughly speaking, a combinatorial structure (a simplicial complex) *K* is built on top of a labeled data set using Delaunay triangulations to, lately, construct a neural network based on a simplicial map defined between *K* and a simplicial complex with just one maximal simplex. This section is devoted to recall some of the basic concepts used in such construction.

The research field of neural networks is exponentially growing and recently, many different architectures, activation functions, and regularization methods have been introduced; thus, it is difficult to find a general definition that covers all the cases. In this paper, we adapt a definition from [20] that fits into our purposes. From now on, n,m,d,k denote positive integers and 〚1,n〛 denote the set of integers {1,…,n}.

**Definition** **1** (adapted from [20]). *A multilayer feedforward network defined between spaces $X\subseteq R^{d}$ and $Y\subseteq R^{k}$ is a function N:X→Y composed of m+1 functions:*

$$N=f_{m+1}\circ f_{m}\circ \cdots \circ f_{1}$$

*where the integer m>0 is the number of hidden layers and, for i∈〚1,m+1〛, the function $f_{i}:X_{i-1}\to X_{i}$ is defined as*

$$f_{i}\left(y\right):=\phi_{i}\left(W^{\left(i\right)};\;y;\;b_{i}\right)$$

*where $X_{0}=X$, $X_{m+1}=Y$, and $X_{i}\subseteq R^{d_{i}}$ for i∈〚1,m〛; $d_{0}=d$, $d_{m+1}=k$, and $d_{i}>0$ being an integer for i∈〚1,m〛 (called the width of the ith hidden layer); $W^{\left(i\right)}\in M_{d_{i-1}\times d_{i}}$ being a real-valued $d_{i-1}\times d_{i}$ matrix (called the matrix of weights of N); $b_{i}$ being a point in $R^{d_{i}}$ (called the bias term); and $\phi_{i}$ being a function (called the activation function). We will call the width of the neural network to the maximum width of hidden layers.*

Throughout this paper, neural networks will be considered as classification models.

**Definition** **2.** *A labeled data set D is a finite set of pairs*

$$D=\left\{\left(p_{j},\ell_{j}\right):\;j\in 〚1,n〛,\;\;p_{j}\in R^{d},\;\ell_{j}\in E^{k}\right\}$$

*where, for j,h∈〚1,n〛, $p_{j}\neq p_{h}$ if j≠h, and $\ell_{j}$ represents a one-hot vector. We say that $\ell_{j}$ is the label of $p_{j}$ or, equivalently, that $p_{j}$ belongs to the class $\ell_{j}$. We will denote by $D_{P}$ the ordered set of points ${\langle p_{j}\rangle }_{j}$.*

Given a data set and a set of neural networks that only differ in their weights, the supervised classification problem consists in finding an available neural network in the set that provides the best classification for the data set. Since neural networks in the set only differ in their weights, finding the best neural network is equivalent to find the best possible weights. Again, several definitions of the concept of supervised classification problem can be provided, mainly depending on the method used to look for the possible weights and the concept of improvement chosen to define the best option.

In this paper, the concept of supervised classification problem for neural networks is defined as follows.

**Definition** **3.** *Given a labeled data set* $D\subset R^{d}\times E^{k}$ *, an integer* m>0 *, and a set of activation functions* $\phi_{i}$ *for* i∈〚1,m〛 *, a supervised classification problem consists of looking for the weights* $W^{\left(i\right)}$ *and bias terms* $b_{i}$ *for* i∈〚1,m〛 *, such that the associated neural network* N:X→Y *, with* $X\subseteq R^{d}$ *,* $Y\subseteq R^{k}$ *and* D⊆X×Y *, satisfies:*

- *N(p)=ℓ for all (p,ℓ)∈D.*
- *N maps x∈X to a vector of scores $N\left(x\right)=\left(y_{1},\ldots ,y_{k}\right)\in Y$ such that $y_{i}\in \left[0,1\right]$ for i∈〚1,n〛 and $\sum_{i\in 〚1,n〛}y_{i}=1$.*

*If such a neural network*N*exists, we will say that*N*characterizes D, or, equivalently, that*N*correctly classifies D*.

The process to search for optimal weights is usually called the training of the neural network. The training most commonly used is based on backpropagation [21]. Nevertheless, in this paper, the optimal weights are not searched through an optimization process. Instead, a combinatorial structure is built on top of the training samples and a function called simplicial map is defined on it; then, a special kind of neural network named simplicial-map neural network is constructed. In order to recall the definition of simplicial-map neural network, we start by recalling the definitions of convex hull and convex polytope.

**Definition** **4.** *The convex hull of a set $S\subset R^{d}$, denoted by conv(S), is the smallest convex set containing S. If S is finite, then conv(S) is called a convex polytope and denoted by P. The set of vertices of a convex polytope P is the minimum set $V_{P}$ of points in P such that $P=\mathrm{conv}(V_{P})$.*

Our construction of simplicial-map neural networks is based on the simplicial complex obtained after a triangulation of the given convex polytope. Let us now recall the concept of simplicial complex.

**Definition** **5.** *Let us consider a finite set V whose elements will be called vertices. A simplicial complex K consists of a finite collection of nonempty subsets (called simplices) of V such that:*

1. *Any subset of V with exactly one point of V is a simplex of K called 0-simplex or vertex.*
2. *Any nonempty subset of a simplex σ is a simplex, called a face of σ.*

*A simplex σ with exactly k+1 points is called a k-simplex. We also say that the dimension of σ is k and write dimσ=k. A maximal simplex of K is a simplex that is not face of any other simplex in K. The dimension of K is denoted by dimK and it is the maximum dimension of its maximal simplices. The set of vertices of a simplicial complex K will be denoted by $K^{\left(0\right)}$. A simplicial complex K is pure if all its maximal simplices have the same dimension.*

An example of simplicial complex is the Delaunay complex defined from the Voronoi diagram of a given finite set of points.

**Definition** **6.** *Let $S=\{p_{1},\ldots ,p_{n}\}$ be a finite set of points in $R^{d}$ in general position. The Voronoi cell $V(p_{i},S)$ is defined as:*

$$V\left(p_{i},S\right):=\left\{x\in R^{d}:\;||x-p_{i}\left|\right|\leq ||x-p_{j}\left|\right|,\;\forall p_{j}\in S\right\}.$$

*The Voronoi diagram of S, denoted as V(S), is the set of Voronoi cells:*

$$V\left(S\right):=\left\{V\left(p_{1},S\right),\ldots ,V\left(p_{n},S\right)\right\}.$$

*The Delaunay complex of S can be defined as:*

$$D\left(S\right):=\left\{\varsigma \subseteq S:\;\cap_{p\in \varsigma }V\left(p,S\right)\neq \emptyset \right\}.$$

The following lemma is just another view of the definition of Delaunay complexes.

**Lemma** **1** (The empty ball property [22] (p. 48)). *Any subset σ⊂S is a simplex of the Delaunay complex of S if and only if it has a circumscribing (open) ball empty of points of S.*

Given d>0, an embedding of a simplicial complex *K* in the *d*-dimensional space $R^{d}$ is usually called a geometric realization of *K*, and it will be denoted by |K|.

One of the key ideas along this paper is that a triangulation can be refined by successive subdivisions of the simplicial complex obtained from the triangulation. There are many different ways to obtain a subdivision of a simplex; in our case, we will use the barycentric subdivision.

**Definition** **7.** *Let K be a simplicial complex with vertices in $R^{d}$. The barycentric subdivision SdK is the simplicial complex defined as follows. The set ${\left(\mathrm{Sd}K\right)}^{\left(0\right)}$ of vertices of SdK is the set of barycenters of all the simplices of K. The simplices of SdK are the finite nonempty collections of ${\left(\mathrm{Sd}K\right)}^{\left(0\right)}$ that are totally ordered by the face relation in K. That is, any k-simplex σ of SdK can be written as an ordered set $\left\{w_{0},\ldots ,w_{k}\right\}$ such that $w_{i}$ is the baricenter of $\mu_{i}$, being $\mu_{i}$ a face of $\mu_{j}\in K$ for i,j∈〚0,k〛 and i<j. In particular, if σ is maximal, then there exists a d-simplex $\{u_{0},\ldots ,u_{d}\}\in K$ satisfying that $w_{i}$ is the barycenter of $\left\{u_{0},\ldots ,u_{i}\right\}$ for i∈〚0,d〛.*

Let us introduce now the notion of simplicial approximation, which is a simplicial map defined on two simplicial complexes *K* and *L* that approximates a given continuous function *g* between the geometric realization of *K* and *L*. First, we recall the concept of vertex maps between two simplicial complexes.

**Definition** **8.** *Given two simplicial complexes K and L, a vertex map $\varphi^{\left(0\right)}:K^{\left(0\right)}\to L^{\left(0\right)}$ is a function from the vertices of K to the vertices of L such that for any simplex σ∈K, the set*

$$\varphi \left(\sigma \right):=\{v\in L^{\left(0\right)}:\;\exists \;u\in \sigma ,\;\varphi^{\left(0\right)}\left(u\right)=v\}$$

*is a simplex of L.*

A vertex map defined on the vertices of a simplicial complex *K* can be linearly extended to a continuous function on the whole simplicial complex *K*.

**Definition** **9.** *The simplicial map $\varphi^{c}:|K|\to \left|L\right|$ induced by the vertex map $\varphi^{\left(0\right)}:K^{\left(0\right)}\to L^{\left(0\right)}$ is a continuous function defined as follows. Let x∈|K|. Then, x∈|σ| for some simplex $\sigma =\{u_{0},\ldots ,u_{k}\}$ of K. So, $x=\sum_{i\in 〚0,k〛}\lambda_{i}u_{i}$ being $\lambda_{i}\geq 0$, for all i∈〚0,k〛 and $\sum_{i\in 〚0,k〛}\lambda_{i}=1$. Then,*

$$\varphi^{c}\left(x\right):=\sum_{i\in 〚0,k〛}\lambda_{i}\varphi^{\left(0\right)}\left(u_{i}\right).$$

Intuitively, a simplicial approximation between two simplicial complex *K* and *L* is a simplicial map that preserves the star of a vertex. Recall that for a vertex *v* of $K^{\left(0\right)}$, the star of *v*, denoted by stv, is the set of simplices of *K* having {v} as a face.

**Definition** **10.** *Let g:|K|→|L| be a continuous function between the geometric realization of two simplicial complexes K and L. A simplicial map $\varphi^{c}:|K|\to \left|L\right|$ induced by a vertex map $\varphi^{\left(0\right)}:K^{\left(0\right)}\to L^{\left(0\right)}$ is a simplicial approximation of g if*

$$g(|\mathrm{st}v|)\subseteq |\mathrm{st}\varphi^{c}\left(v\right)|$$

*for each vertex v of $K^{\left(0\right)}$.*

Next, the main definition used in this paper is recalled. Given a simplicial map between the geometric realizations of two finite pure simplicial complexes, a two-hidden-layer feedforward network can be built. Such neural network is called a simplicial-map neural network and the value of its weights can be exactly computed from the vertex map associated to the simplicial map. In other words, there is no need to train the neural network to find the optimal weights.

**Definition** **11.** *Let K and L be two finite pure simplicial complexes of dimension d and k, respectively. Let us consider the simplicial map $\varphi^{c}:|K|\to \left|L\right|$ induced by a vertex map $\varphi^{\left(0\right)}:K^{\left(0\right)}\to L^{\left(0\right)}$. Let $\left\{\sigma_{1},\ldots \sigma_{n}\right\}$ be the maximal simplices of K, where $\sigma_{i}=\left\{u_{0}^{i},\ldots ,u_{d}^{i}\right\}$ and $u_{h}^{i}\in R^{d}$ for i∈〚1,n〛 and h∈〚0,d〛. Let $\left\{\mu_{1},\ldots ,\mu_{m}\right\}$ be the maximal simplices of L, where $\mu_{j}=\left\{v_{0}^{j},\ldots ,v_{k}^{j}\right\}$ and $v_{h}^{j}\in R^{k}$ for j∈〚1,m〛 and h∈〚0,k〛. The simplicial-map neural network induced by $\varphi^{c}$, denoted by $N_{\varphi }$, is the two-hidden-layer feedforward neural network having the following architecture:*

- *an input layer with $d_{0}=d$ neurons;*
- *a first hidden layer with $d_{1}=n\left(d+1\right)$ neurons;*
- *a second hidden layer with $d_{2}=m\left(k+1\right)$ neurons; and*
- *an output layer with $d_{3}=k$ neurons.*

*This way, $N_{\varphi }=f_{3}\circ f_{2}\circ f_{1}$ being $f_{i}\left(y\right)=\phi_{i}\left(W^{\left(i\right)};\;y;\;b_{i}\right)$, for i∈〚1,3〛, defined as follows. First, $W^{\left(1\right)}=\left(\begin{array}{c} W_{1}^{\left(1\right)}\\ \vdots \\ W_{n}^{\left(1\right)} \end{array}\right)\in M_{n(d+1)\times d}$ and $b_{1}=\left(\begin{array}{c} B_{1}\\ \vdots \\ B_{n} \end{array}\right)\in R^{n(d+1)}$ where*

$$\left(\begin{array}{ccc} W_{i}^{\left(1\right)} & | & B_{i} \end{array}\right)={\left(\begin{array}{ccc} u_{0}^{i} & \ldots & u_{d}^{i}\\ 1 & \ldots & 1 \end{array}\right)}^{-1}\in M_{\left(d+1)\times (d+1\right)}$$

*being $W_{i}^{\left(1\right)}\in M_{(d+1)\times d}$ and $B_{i}\in R^{d+1}$. The function $\phi_{1}$ is defined as*

$$\phi_{1}\left(W^{\left(1\right)};y;b_{1}\right):=W^{\left(1\right)}y+b_{1}.$$

*Second, $W^{\left(2\right)}=\left(W_{h,\ell }^{\left(2\right)}\right)\in M_{m(k+1)\times n(d+1)}$$b_{2}\in R^{m(k+1)}$ is null where*

$$W_{h,\ell }^{\left(2\right)}:=\left\{\begin{array}{cc} 1 & if\;\varphi^{\left(0\right)}\left(u_{t}^{i}\right)=v_{r}^{j},\\ 0 & otherwise; \end{array}\right.$$

*being h=j(r+1) and ℓ=i(t+1) for i∈〚1,n〛; j∈〚1,m〛; t∈〚0,d〛; and r∈〚0,k〛. The function $\phi_{2}$ is defined as:*

$$\phi_{2}\left(W^{\left(2\right)};y;b_{2}\right):=W^{\left(2\right)}y.$$

*Thirdly, $W^{\left(3\right)}=\left(\begin{array}{ccc} W_{1}^{\left(3\right)} & \ldots & W_{m}^{\left(3\right)} \end{array}\right)\in M_{k\times m(k+1)}$ and $b_{3}\in R^{k}$ is null being*

$$W_{j}^{\left(3\right)}:=\left(\begin{array}{ccc} v_{0}^{j} & \cdots & v_{k}^{j} \end{array}\right)\;forj\in 〚1,m〛.$$

*The function $\phi_{3}$ is defined as:*

$$\phi_{3}\left(W^{\left(3\right)};y;b_{3}\right):=\frac{\sum_{j\in 〚1,\ell 〛}z^{j}\psi \left(y^{j}\right)}{\sum_{j\in 〚1,\ell 〛}\psi \left(y^{j}\right)}$$

*being $z^{j}:=W_{j}^{\left(3\right)}y^{j}$ for $y=\left(\begin{array}{c} y^{1}\\ \vdots \\ y^{m} \end{array}\right)\in M^{m\cdot (k+1)}$ and*

$$\psi \left(y^{j}\right):=\left\{\begin{array}{cc} 1 & if\;all\;the\;coordinates\;of\;y^{j}\;are\geq 0,\\ 0 & otherwise. \end{array}\right.$$

As shown in [17], simplicial-map neural networks can be used for classification purposes. Given a labeled data set $D\subset R^{d}\times R^{k}$, we first compute a convex polytope P surrounding *D*. Second, we compute the Delaunay complex K=D(S) of the set $S=D_{P}\cup V_{P}$ and define a simplicial complex *L* composed of a maximal simplex $\sigma =\{v_{0},\ldots ,v_{\ell }\}$ such that its dimension is equal to the number of classes of *D*. Finally, a vertex map that induces a simplicial-map neural network that correctly classifies *D* is defined as follows (see Proposition 4 in [17]):

(1) $$\varphi^{\left(0\right)}\left(u\right):=\left\{\begin{array}{cc} v_{i} & \;\mathrm{if}\;(u,i)\in D,\\ v_{0} & \;\mathrm{if}\;u\in V_{P}. \end{array}\right.$$

However, this simplicial-map neural network is not robust to adversarial attacks as shown in Proposition 5 in [17]. To construct simplicial-map neural networks robust to adversarial attacks of a given bounded size, the idea is to define a width decision boundary through barycentric subdivisions. Nevertheless, with each barycentric subdivision iteration, the number of simplices grows as it is claimed in Remark 1 of [16].

Once we have introduced all the necessary notions to explicitly construct a neural network to solve a classification problem, we present a methodology to reduce the size of such a network without hindering its performance.

## 3. Description of the Methodology

In this section, we propose a methodology to reduce the size of a simplicial-map neural network used for classification tasks.

Recall that given a labeled data set *D* with *k* classes, the process to obtain a simplicial-map neural network that correctly classifies *D* is: (1) to compute a convex polytope P surrounding *D*; (2) to compute the Delaunay complex *K* of the set $D_{P}\cup V_{P}$; (3) to compute a vertex map $\varphi^{\left(0\right)}$ from the vertices of *K* to the vertices of a simplicial complex *L* with only one maximal *k*-simplex; and (4) to compute a simplicial-map neural network $N_{\varphi }:|K|\to \left|L\right|$, from the simplicial map $\varphi^{c}$.

However, this simplicial-map neural network $N_{\varphi }$, as many other neural networks, can suffer the attack of adversarial examples. In [17], a method to increase the robustness of the simplicial-map neural network to such attacks was developed by applying successive barycentric subdivisions to *K* and *L* depending on the desired robustness. However, the iteration of barycentric subdivisions results in the exponential growth of the number of simplices. Therefore, the storage and computational cost of the simplicial map $\varphi^{c}$ and the simplicial-map neural network $N_{\varphi }$ grow exponentially.

In order to avoid this problem, in this paper, we propose a method to reduce the storage and computational cost of the simplicial-map neural network $N_{\varphi }:|K|\to \left|L\right|$ by removing points of the given labeled data set *D* but keeping exactly the same accuracy as $N_{\varphi }$. The idea is to remove those simplices from *K* whose vertices belong all to the same class. Therefore, those simplices with vertices in the decision boundary remain, leaving the decision boundary invariant.

Let us now formalize this idea. Let $D=\left\{\left(p_{j},\ell_{j}\right):\;j\in 〚1,n〛,\;\;p_{j}\in R^{d},\;\ell_{j}\in E^{k}\right\}$ be a data set and let $N_{\varphi }$ be the simplicial-map neural network obtained using the process described above. Our aim is to obtain a subset $\tilde{D}$ that induces a simplicial-map neural network ${\tilde{N}}_{\tilde{\varphi }}$ with exactly the same behavior than $N_{\varphi }$. The procedure is described in Algorithm 1.

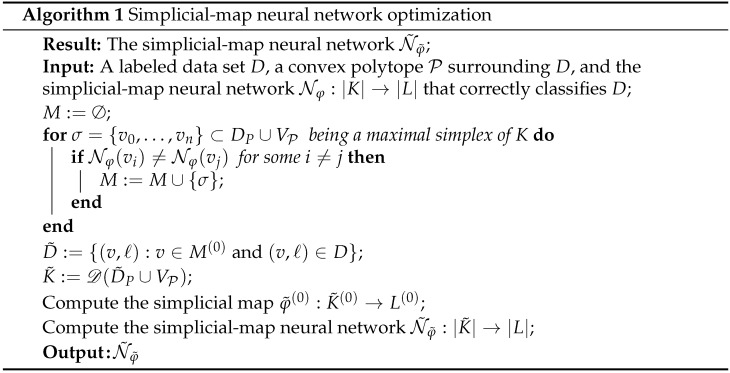



In Section 4, using a high-dimensional data set composed of digit images, we check experimentally that both simplicial-map neural networks ${\tilde{N}}_{\tilde{\varphi }}$ and $N_{\varphi }$ have the same behavior. The following partial result also supports that idea.

**Lemma** **2.** *Let D be a labeled data set, let $N_{\varphi }:|K|\to \left|L\right|$ be the simplicial-map neural network that correctly classifies D, constructed following the method given in [17], and let ${\tilde{N}}_{\tilde{\varphi }}$ be the simplicial-map neural network obtained from Algorithm 1. If $\sigma =\{v_{0},\ldots ,v_{n}\}\in K$ satisfies that $N_{\varphi }\left(v_{i}\right)\neq N_{\varphi }\left(v_{j}\right)$ for some i≠j, then ${\tilde{N}}_{\tilde{\varphi }}\left(x\right)=N_{\varphi }\left(x\right)$ for all x∈|σ|.*

**Proof.**  Let $\sigma =\{v_{0},\ldots ,v_{n}\}$ be a simplex of *K* such that $N_{\varphi }\left(v_{i}\right)\neq N_{\varphi }\left(v_{j}\right)$ for some i≠j. Then, σ is a face of a maximal simplex μ of *K* with all its vertices belonging to ${\tilde{D}}_{P}\cup V_{P}$. Therefore, μ is a maximal simplex of $\tilde{K}$ (by Lemma 1) and ${\tilde{N}}_{\tilde{\varphi }}\left(x\right)=N_{\varphi }\left(x\right)$ for any x∈|μ|. Since σ is a face of μ then ${\tilde{N}}_{\tilde{\varphi }}\left(x\right)=N_{\varphi }\left(x\right)$ for any x∈|σ|. □

**Figure 1:**
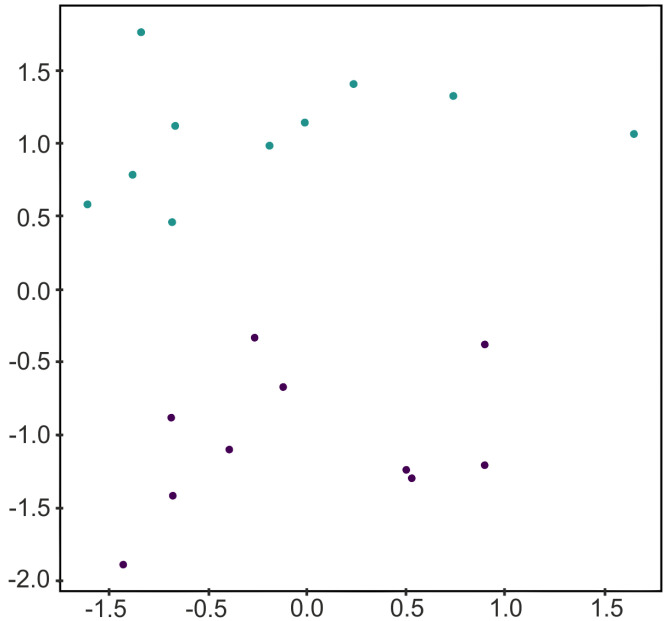
A toy example of a two-dimensional data set for binary classification generated using the scikit-learn package implementation of [23].

**Figure 2:**
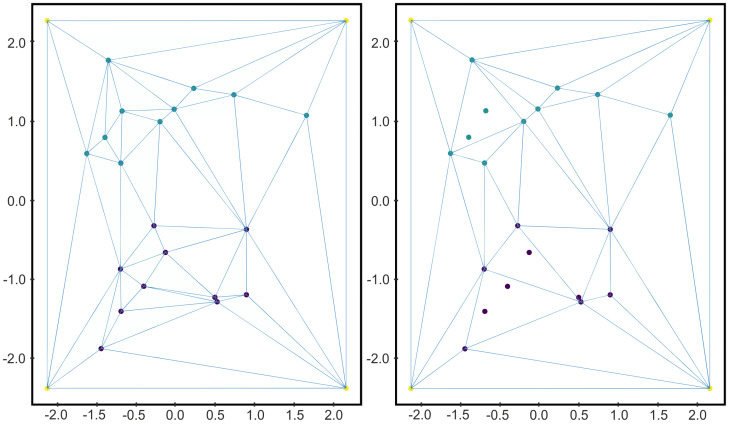
On the left, the Delaunay triangulation of the labeled data set *D* given in Figure 1 together with the vertices of the square polytope surrounding *D*. On the right, the Delaunay triangulation of a subset of *D* obtained as described in Algoritm 1. As we can see, the triangles whose vertices belonged all to the same class disappeared.

In order to illustrate Algorithm 1, let us consider the two-dimensional labeled data set *D* given in Figure 1. Let us consider a square surrounding the data set as the convex polytope P, and let us compute the Delaunay complex $K=D(D_{P}\cup V_{P})$ as shown in Figure 2. Then, *K* is composed of 24 points and 42 2-simplices. Applying Algorithm 1 is equivalent to remove those 2-simplices of *K* whose vertices belong, all of them, to the same class. Then, we consider only the vertices of the surviving 2-simplices and the Delaunay complex is computed again. In that case, the resultant simplicial complex is composed of 18 points and 30 2-simplices (see Figure 2).

**Lemma** **3.** *If the points of $D_{P}\cup V_{P}$ are in general position, then the reduced simplicial neural network ${\tilde{N}}_{\tilde{\varphi }}$ can always be computed from Algorithm 1.*

**Proof.**  If the points of $D_{P}\cup V_{P}$ are in general position, then any subset of points of $D_{P}\cup V_{P}$ are in general position, so the the Delaunay triangulation of ${\tilde{D}}_{P}\cup V_{P}$ can always be computed, as well as the simplicial-map neural network ${\tilde{N}}_{\tilde{\varphi }}$. □

**Figure 3:**
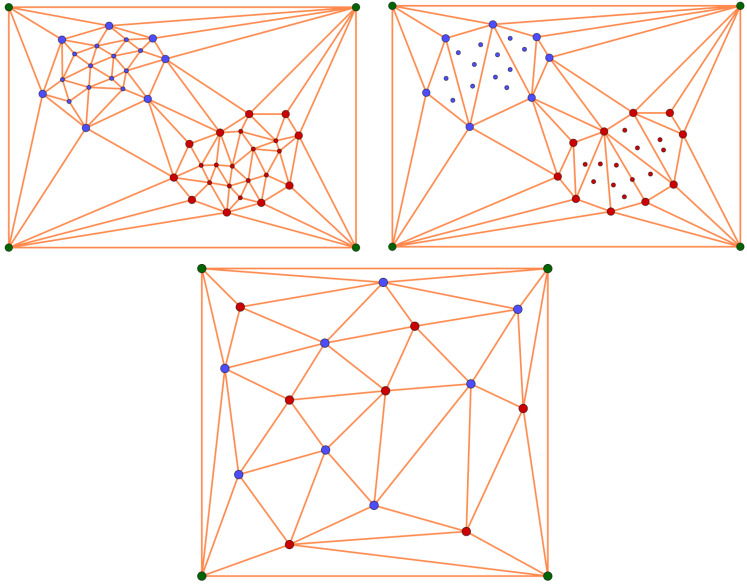
From left to right and from top to bottom: (1) a separable and dense binary data set *D*; (2) the data set obtained after applying Algorithm 1 to *D*; (3) a data set that cannot be reduced by applying Algorithm 1.

Let us notice that, depending on the distribution of the data set, the reduction obtained after applying Algorithm 1 can be significant or not. Specifically, if the different classes of *D* are not mixed, then we can expect good results of Algorithm 1. The reduction will be optimum when the data set is separable and dense. In such case, most of the simplices would have vertices of the same class and be removed when Algorithm 1 is applied. An example of these two opposite cases are shown in Figure 3.

## 4. Experiments

**Figure 4:**
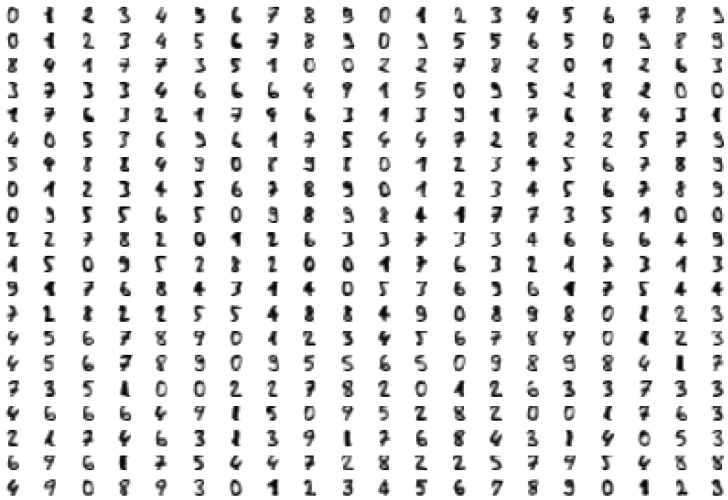
Some of the 1797 images used in the experiment. The images are labeled from 0 to 9 in a natural way. Each image is grey-scaled and has 8×8 pixels, so it can be represented as a point in $R^{64}$. In order to visualize such 1797 64-dimensional points, $R^{64}$ has been projected into $R^{2}$ using the UMAP algorithm. Figure 5 shows the projection on $R^{2}$ of the 1797 images.

**Figure 5:**
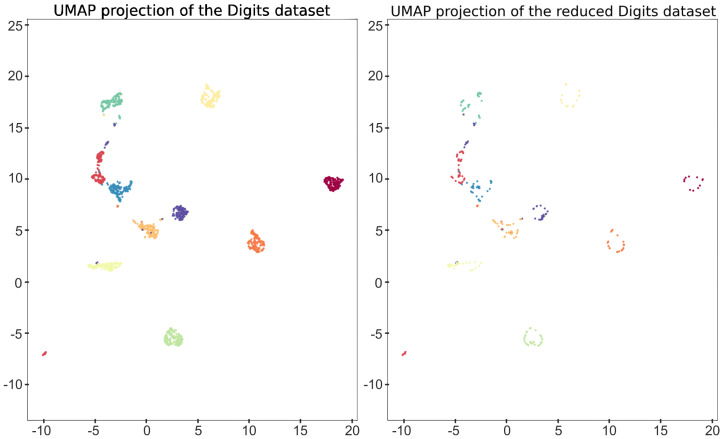
Visualization of the UMAP 2D representation of the original data set used (**left**), and the simplified data set obtained (**right**).

In this section, a high-dimensional data set composed of digit images is considered. In this case, for visualization purposes, the data set is firstly embedded to obtain a low-dimensional representation using the UMAP algorithm [24]. The data set is composed of 1797 greyscale images of dimension 8×8. These images represent digits from 0 to 9. In Figure 4, some of the images are shown and, in Figure 5, the two-dimensional UMAP output is displayed, representing the full data set. In order to illustrate our method by providing a graphical intuition, we will focus on the 2D representation of the digits data set, but the construction can be conducted with any dimension of the input.

**Figure 6:**
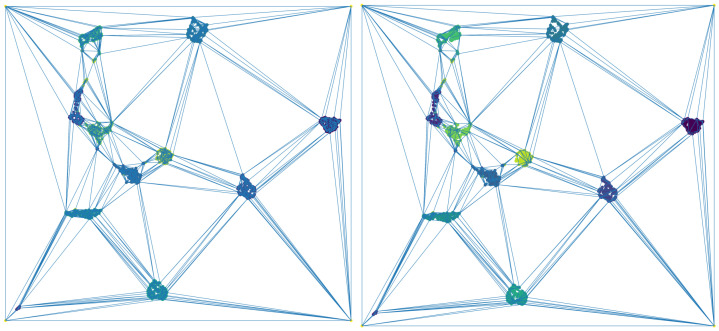
On the (**left**), the Delaunay triangulation of the original data set and, on the (**right**), the Delaunay triangulation of the simplified data set.

Let us focus on the 1797 two-dimensional points of the UMAP representation of the digits data set *D* depicted in Figure 5, and let us consider a square P surrounding such a cloud of points $D_{P}$. According to [17], a simplicial-map neural network $N_{\varphi }$ can be built in order to correctly classify *D*. Now, let us apply Algorithm 1 to obtain a simplified version of $N_{\varphi }$ that also correctly classify *D*. This way, all of the points in $D_{P}$ surrounded by points belonging to the same class were removed to obtain a reduced data set $\tilde{D}$ inducing the same simplicial-map neural network than *D*. In Figure 5, the two-dimensional representation of the reduced data set is shown. The next step is the computation of the Delaunay triangulation using the data set $\tilde{D}$ and the vertices of the square P. In Figure 6, the Delaunay triangulation is shown for both the original and the simplified data set. The Delaunay triangulation of the original data set is composed of 3596 2-simplices, whereas the Delaunay triangulation of the simplified data set is composed of 604 2-simplices and 305 points reaching a remarkable reduction in the number of simplices. The results are summarized in Table 1. Finally, the induced simplicial-map neural networks were experimentally compared obtaining exactly the same performance.

Lastly, Algorithm 1 was experimentally tested for synthetically generated two- and three-dimensional data sets. The numerical results can be found in Table 2 and Table 3, respectively. Let us point out that in the three-dimensional data set with a greater amount of points, the reduced data set has a reduction of approximately 73%, inducing the same simplicial-map neural network.

The code of the experimentation can be consulted in https://github.com/Cimagroup/DelaunayTriangAndNN (accessed on 30 August 2021).

## 5. Conclusions

Simplicial-map neural networks are a recent neural network architecture based on simplicial maps defined between a triangulation of the given data set and a simplicial complex encoding the classification problem. These neural networks are refined by applying barycentric subdivisions to ensure their robustness. The iterative application of barycentric subdivisions increases the number of simplices exponentially. Therefore, the width of the neural network also increases exponentially. In this paper, we have provided a way to reduce the number of simplices but maintaining the performance of the neural network. The proposed method has been experimentally tested. As further work, we plan to formally prove that our optimized simplicial-map neural network ${\tilde{N}}_{\tilde{\varphi }}$ is equivalent to the original one $N_{\varphi }$.

## Figures and Tables

**Table 1:** The size of the data set used in the digits experiment, the number of 2-simplices obtained after computing the Delaunay triangulation, and the ones obtained after applying Algorithm 1.

Data Set Size	2-Simplices	2-Simplices (Reduced)	Data Set Size (Reduced)
1801	3596	604	305

**Table 2:** The size of the two-dimensional synthetic data sets used, the number of 2-simplices obtained after computing the Delaunay triangulations, and the ones obtained after applying Algorithm 1.

Data Set Size	2-Simplices	2-Simplices (Reduced)	Data Set Size (Reduced)
14	22	22	14
104	202	58	32
1004	2002	230	118
10,004	20,002	8384	4195
100,004	200,002	6620	3313
1,000,004	2,000,002	73,488	36,747

**Table 3:** The size of the three-dimensional synthetic data sets used, the number of 2-simplices obtained after computing the Delaunay triangulations, and the ones obtained after applying Algorithm 1.

Data Set Size	3-Simplices	3-Simplices (Reduced)	Data Set Size (Reduced)
14	34	29	13
104	551	391	75
1004	6331	1647	272
10,004	66,874	30,357	4556
100,004	672,097	147,029	21,955
1,000,004	6,762,603	1,858,204	274,635

## Data Availability

The code and data of the experimentation can be consulted in https://github.com/Cimagroup/DelaunayTriangAndNN.
